# International chemical identifier for reactions (RInChI)

**DOI:** 10.1186/s13321-018-0277-8

**Published:** 2018-05-09

**Authors:** Guenter Grethe, Gerd Blanke, Hans Kraut, Jonathan M. Goodman

**Affiliations:** 1 14133 Mountain View Ct, Poway, 92064 CA USA; 2 StructurePendium Technology GmbH, Essen, Germany; 3 InfoChem Gesellschaft für Chemische Information mbH, Munich, Germany; 4 Department of Chemistry, University of Cambridge, Lensfield Road, Cambridge, CB2 1EW UK

## Abstract

The Reaction InChI (RInChI) extends the idea of the InChI, which provides a unique descriptor of molecular structures, towards reactions. Prototype versions of the RInChI have been available since 2011. The first official release (RInChI-V1.00), funded by the InChI Trust, is now available for download (http://www.inchi-trust.org/downloads/). This release defines the format and generates hashed representations (RInChIKeys) suitable for database and web operations. The RInChI provides a concise description of the key data in chemical processes, and facilitates the manipulation and analysis of reaction data.

## Background

Since its inception, the IUPAC International Chemical Identifier (InChI) [1, 2] has found wide acceptance as a standard in the chemical community. In order to widen the applicability of the identifier, the IUPAC Division VIII Subcommittee and the InChI Trust [3] have initiated several projects to extend the usage of the identifier. Among these is the development of a non-proprietary, international identifier for reactions (RInChI) [4] to describe chemical reactions in a unique machine-readable character string based on the InChI algorithm suitable for data storage and indexing. For this purpose, a working group was established in 2008, which produced a series of prototypes at the University of Cambridge [5, 6]. These prototypes were tested on thousands of trial reactions, and, ultimately, a database of about a million reactions was assembled, using data that NextMove software has extracted from the patent literature.

**Fig. 1:**
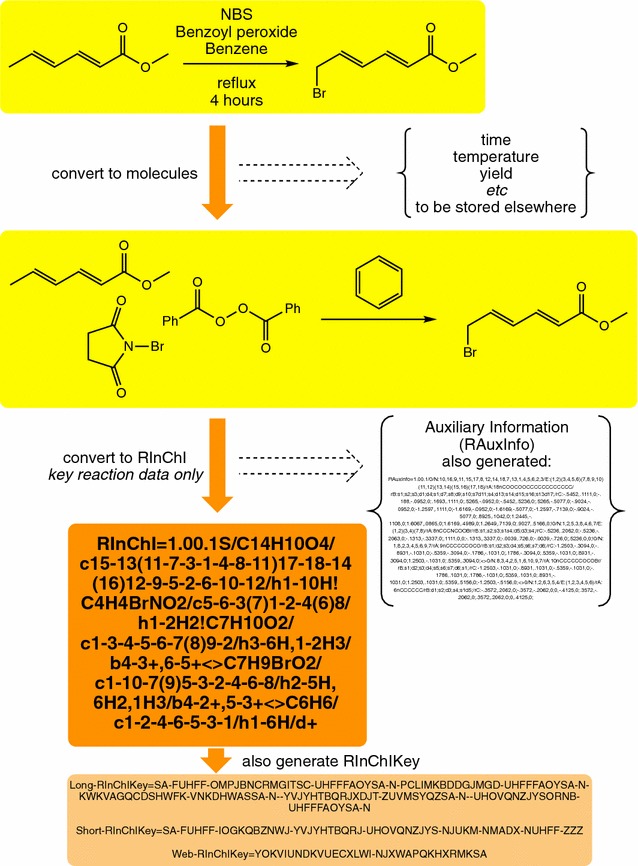
An example of the RInChI generation process [9]

In this paper, we outline the process for the construction of RInChIs and the software that is available to automate this process. It is straightforward to construct a few RInChIs by hand, using the standard InChI generation software. The generation of thousands of RInChIs is much better done using the program outlined here and available for download from the InChI Trust website [3]. The software also generates RInChIKeys, which are hashed versions of RInChIs, and auxiliary information which can be helpful in regenerating detailed reaction descriptions. An example of the process is shown in Fig. 1.

## Introduction

Developing a description of reactions that is mutually intelligible, unambiguous and useful requires the development of a new language with the right balance of clarity and evocative imprecision. A description that contained all of the available information about a chemical transformation would, necessarily, be complicated and long. Such detail is important, but unstructured data cannot be easily ordered, searched and analysed. A very brief outline or label would be straightforward to handle, and could be used as an index to a fuller description. If different people, working on the same reaction, could generate the same label without conferring, then the labels would be powerful tools for ordering chemical information. How could such a label be constructed?

This is not a new problem. Developing a de facto standard language, during a time when communication and data transfer were being revolutionized and becoming more powerful and faster than ever before, was a challenge for Geoffrey Chaucer, the fourteenth century astronomer, poet and the author of the *Canterbury Tales*. He wrote an instruction manual for a device that quantitatively measured the position of the stars. He also described the world in a new language, so well that his influence is still felt. He recorded it with insight and inspiration that enabled subsequent writers to express themselves more clearly. How would the modern world be different if the language of so much of science had different words and concepts?

An advance in technology, the invention of the printing press, gave his work wide availability. His genius for writing, and his perceptive observations, led to his influence and the widespread adoption of his use of language. Chaucer defined terms that were re-used because of their explanatory power.

When Chaucer refers to gold, his meaning is close to the modern implications of the word, but even gold is not really gold. Chaucer’s gold would have been a mixture of elements, in a composition unknown and perhaps now unknowable. We can only describe the things for which we have words. Substances are hard to describe; transformations much harder. How can they be described? “If gold rust, what shall iron do?”

This is an immediate problem for chemistry: how can we describe chemical transformations? Millions of chemical reactions have been studied, illuminated by our current understanding of molecular structure, but this information is stored in inconsistent language and often bound by publishers’ license conditions. If we could take these data and use them together, we could discover far more than is possible focussing on small groups of reactions, or individual texts. Different researchers, working in different places, need to create the same label for the same reaction, and so communicate effectively with each other.

As data becomes more accessible through open university repositories and other freely-available resources, the drivers for learning and communicating on a global level become more powerful. We just need consistent, mutually intelligible, language. The RInChI formalism is an attempt to provide this.

Reactions are more difficult to codify than molecules. A molecule can usually be uniquely and precisely defined by a structural drawing, which shows the atom types, the bond connectivity, and the stereochemistry. A reaction includes molecules, but also times, temperatures, concentrations, rates of mixing, yields, and numerous other quantities that are recorded with varying levels of precision. It is unlikely that two research groups, independently working on similar reactions, will ever carry out exactly the same reaction, under precisely the same conditions, and get identical results. There will almost always be small differences in concentrations, times, and yields, even if the same starting materials are used and the same products are obtained. A full description of a reaction necessarily includes a large amount of information. Organic chemistry journals have requirements for exactly what data needs to be included for publication. However, it is widely appreciated that even these data are not always enough for another research group to reproduce the reaction.

One approach to this issue is to include more data, and this is reflected in the development of standards for publications in this field. However, it is also useful to do the opposite: including less data. This will make it harder to reproduce the reactions, but will make it easier to check if different researchers are doing essentially the same reaction despite differences in scale, solvent, etc. A Wittig reaction, for example, is a useful label for a process that has a very large number of variants. A reaction identifier that had enough detail to be useful and yet not so much that practically identical processes would be given different labels, would be extremely useful in the creation and analysis of reaction databases. The Reaction Inchi (RInChI) was developed with this in mind.

## Results and discussion

### RInChI content

What is the heart of a chemical reaction? The starting materials are critical, as are the products, and any catalysts or other substances present at both the beginning and at the end of a reaction, which we call an agent. Almost no other information is required to construct a RInChI. Indeed, not all of this information is needed. A half-reaction, such as a RInChI with starting materials and no identified products, is a valid RInChI.

A central focus of designing the RInChI was that the barrier to its use should be as low as possible, whilst still capturing useful information. For this reason, no knowledge of a reaction’s mechanism is required to construct a RInChI. Such a requirement would make it harder to generate a valid RInChI, and so reduce their utility. It would also mean that people sometimes created RInChIs with the best of intentions, but which contained misleading information. Keeping the RInChI as simple as possible should maximize use and minimize erroneous records.

The RInChI contains slightly more information than this. Most reactions have a direction, but many reactions are reversible. A change to concentration or temperature may make a reaction run backwards. For this reason, the direction of a reaction is recorded as a flag towards the end of the RInChI, so this information can be readily identified and discarded when required. If two different researchers do the same reaction with slightly different conditions, so they come to opposite conclusions about the direction of the reaction, they will both generate RInChIs that are identical, except for the direction flag. A search of a database that neglected direction would find both reports.

RInChIs use InChIs to describe molecules. The InChI is a general-purpose molecular identifier which is very widely used and which can give a unique, interpretable label to most organic molecules. The InChI has a few limitations, which are likely to be addressed in future versions of the InChI standard. RInChIs use the InChI standard version 1.04. As new versions of InChI are released, they are expected to replace version 1.04 in the RInChI. For this reason, RInChIs do not attempt to address any of the issues that will be solved by InChIs themselves, in due course.

Many reactions, however, contain substances that cannot be described by InChIs. Structureless materials, in the sense of materials for which there is no InChI, include heterogeneous metals, organometallic compounds, enzymes and natural substances such as linseed oil. The RInChI does not attempt to identify such mysterious entities, but it does track how many “no-structure” materials are present in the starting materials, the products and the agents.

The RInChI includes these and nothing else. RInChI does not describe reaction details such as yield, temperature, concentration, pressure, time, exotherms, phase changes, appearance, mixing, etc. Further information may be stored in RAuxInfo or in other auxiliary information layers.

## Methods

### Constructing a RInChI

RInChI Version 1.00 comprises six layers, which may readily be constructed by hand, provided that a program to generate InChI standard version 1.04 is available. The automated process for doing this will be described in §5.1. The layers are separated by a solidus symbol, commonly called “slash” (“/”), except for the layers which list molecules that are separated by “less than” and “greater than” signs: “<>”.

#### RInChI layer one

The first layer defines the RInChI version, and is the same for all V1.00 RInChIs. It includes the version number (1.00) followed by “1S” indicating that the standard InChI calculator was used. For all the examples in this paper, the first layer is: RInChI=1.00.1S.

#### RInChI layers two and three

The second and third layers list the reactants and the products of the reaction, represented by InChIs, omitting the first layer that is the same for all of them. Molecules are separated by an exclamation mark “!” and the layers are separated by “<>”. The InChIs are listed alphabetically within each layer. Because the RInChI distinguishes starting materials and products by a flag in layer five, and not by the order or layers two and three, these two layers are in alphabetical order. Layer two will always be before layer three when sorted alphabetically. RInChIs with no molecules listed in either layer two or layer three, or if both layers are empty, are still valid RInChIs, indicating either that these components are unknown, or else that they contain no-structure materials, about which there is more information in layer six.

No molecule may appear in both layer two and layer three, and no molecule should be repeated within each layer. Anything that is in both layers should be removed from both and added to layer four.

#### RInChI layer four

The fourth layer comprises catalysts, solvents and anything else that is present both at the beginning and the end of the reaction. We call such materials *agents*. There may be uncertainty about whether a solvent is a reagent or inert whilst the reaction goes on without it. In both cases, the solvent can be called an *agent* rather than requiring this mechanistic detail to be pinned down before a RInChI can be constructed.

#### RInChI layer five

The fifth layer indicates whether layer two or layer three represents the starting materials. It is separated from layer four by a solidus, and is the letter “d” followed by a plus sign “+”, where layer two represents the starting materials, a minus sign “−”, if layer three represents the starting materials, of an equals sign “=” if the reaction is regarded as an equilibrium. If the fifth layer is empty, the direction of the reaction is unspecified.

#### RInChI layer six

The sixth layer contains information about no-structure materials, listing the number of distinct no-structure substances in layers two, three and four. The format is the letter “u” followed by a number indicating the total no-structure materials in each layer. For example, a RInChI with two no-structure materials in layer two, three in layer three, and four in layer four, would have a sixth layer: /u2-3-4.

Omitting the sixth layer implies there are no known no-structure materials. Omitting the final section (e.g. /u2-3) implies there are no no-structure agents.

#### Example

A complete RInChI, therefore, looks something like this:

- **RInChI=1.00.1S** /layer2<>layer3<>layer4/d+/u2-0-1

Everything can be omitted, except the characters in bold; separators are omitted if they have nothing to separate.

**Fig. 2:**
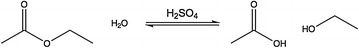
The hydrolysis of ethyl acetate

Figure 2 shows a simple equilibrium reaction: the hydrolysis of ethyl acetate. The six layers of the RInChI may be readily constructed. Layer one will be the standard: RInChI=1.00.1S.

The next layers contain molecules. There are five different molecules, all of which have an InChI:

Ethyl acetate: InChI=1S/C4H8O2/c1-3-6-4(2)5/h3H2,1-2H3
Water: InChI=1S/H2O/h1H2
Sulfuric acid: InChI=1S/H2O4S/c1-5(2,3)4/h(H2,1,2,3,4)
Acetic acid: InChI=1S/C2H4O2/c1-2(3)4/h1H3,(H,3,4)
Ethanol: InChI=1S/C2H6O/c1-2-3/h3H,2H2,1H3

The “InChI=1S/” is omitted from InChIs listed in the RInChI as it is already specified in layer one. Layer four contains only sulfuric acid, so it will be: H2O4S/c1-5(2,3)4/h(H2,1,2,3,4).

To decide on layers two and three, we have to sort the relevant InChIs alphabetically. So, ethyl acetate and water will be one group; acetic acid and ethanol the other:

- C4H8O2/c1-3-6-4(2)5/h3H2,1-2H3
- H2O/h1H2
- C2H4O2/c1-2(3)4/h1H3,(H,3,4)
- C2H6O/c1-2-3/h3H,2H2,1H3

“C2H4” comes before “C4H8” and so acetic acid and ethanol are layer two; ethyl acetate and water are layer three. This is the opposite way around to the diagram, but the RInChI does not record this detail of the drawing. This means that the arbitrary choices made by the people who draw the reaction are removed and only the essence remains. The reaction is the same whether the ethanol is to the left or the right of the acetic acid, and the RInChI does not record this. Layers two, three and four, therefore, are:

- C2H4O2/c1-2(3)4/h1H3,(H,3,4)!C2H6O/c1-2-3/h3H,2H2,1H3<>
- C4H8O2/c1-3-6-4(2)5/h3H2,1- 2H3!H2O/h1H2<>
- H2O4S/c1-5(2,3)4/h(H2,1,2,3,4)

Layer five indicates the reaction is an equilibrium: d=

Layer six is omitted, because all the molecules involved have structures. The complete RInChI is:
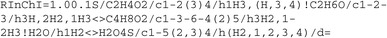


The RInChI is one long string. The line breaks are necessary only to show the whole thing in a standard font size.

### RInChIKeys

RInChI can become rather long. This can be addressed by hashing the RInChI into a RInChIKey, a process which can be done in a number of different ways. Is it better to keep information about the individual molecules, to keep information about the layers, or to simplify even more? Shorter keys are easier to handle, but contain less information. The version 1.00 release of the RInChI code provides three options containing successively less information: Long-RInChIKeys, Short-RInChIKeys and Web-RInChIKeys. These cannot be easily calculated by hand, and so the best way to generate them is to use the program. For all the keys, clashes are likely to be exceedingly infrequent, but it is important to bear in mind that they are possible.

#### Long-RInChIKey

Long-RInChIKeys are a valuable tool for the database storage of reactions. Beside uniqueness checks, they allow the identification of each reaction component by simple text searches based on Standard InChIKeys. Any molecule within a reaction can be identified by its InChIKey, which allows exact molecule searches based on text searches for InChIs within any reaction databases containing the long RInChIKey. In addition, each InChIKey from the long RInChIKey can be easily used to build synthesis trees.

The long RInChIKey does not, however, have a fixed length and it may be rather long for a reaction containing many components. The long RInChIKey for the reaction in Fig. 2 is:
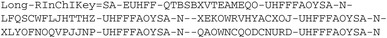


“SA” at the beginning of the key indicates that this was generated with a standard InChI (“S”) and with version 1.04 of the InChI software (“A”). The first letter of the next block “E” indicates that this is an equilibrium. “F”, “B” and “U” are also possible here, indicating Forward (“d+”), Backward (“d−“) or Undefined. The next four letters, “UHFF”, are unused. The rest of the Long-RInChIkey lists the molecules in the reaction, with a double-hyphen “–” separating layers two, three and four. The repeated “uhfffaoysa” indicates that all the components lack stereochemistry. This can be decoded by comparing it with the InChIKey for each component:

Acetic acid: QTBSBXVTEAMEQO-UHFFFAOYSA-N
Ethanol: LFQSCWFLJHTTHZ-UHFFFAOYSA-N
Ethyl acetate: XEKOWRVHYACXOJ-UHFFFAOYSA-N
Water: XLYOFNOQVPJJNP-UHFFFAOYSA-N
Sulfuric acid: QAOWNCQODCNURD-UHFFFAOYSA-N

In this example, the Long-RInChIKey is slightly longer than the original RInChI. If the reaction had contained more complex molecules, however, each InChIKey would be rather shorter than the corresponding InChI, and so conversion to the Long-RInChIKey would reduce the overall length of the RInChI.

This key is likely to be useful for searching for individual molecules in a compact way. It can be desirable to have a fixed-length key, even though this is likely to lose more information. This is available with the Short-RInChIKey.

#### Short-RInChIKey

Hashing over all major layers of the InChI in layers two, three and four, and then hashing all minor layers and protonation states, generates the Short-RInChIKey. The Short-RInChIKey has a fixed length of 55 letters, plus eight hyphens to separate the components, resulting in a total of 63 characters. The fixed length of Short-RInChIKey makes it suitable for exact searches of reactions in databases, indexing reactions in databases and linking identical reactions in different databases. The Short-RInChIKey for the reaction in Fig. 2 is:
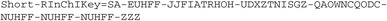


Like the Long-RInChIKey, the “SA” at the beginning indicates the use of the Standard InChI and the version of RInChI being used. The next component “EUHFF” also corresponds exactly to the Long-RInChIKey. The next three ten-character components correspond to RInChI layers two, three and four, respectively, of the original RInChI. For each one, the major layers of the component InChI are all hashed together into a single ten-character string, in the same order as in the RInChI. “JJFIATRHOH”, therefore, corresponds to the major InChI layers of acetic acid and ethanol, hashed together, producing a string which is compact through a process which is, for most practical purposes, irreversible. If any of the RInChI layers are empty, they produce the string “UHFFFADPSC”.

The next components are three groups of five letters, which are generated from the total protonation states and the minor InChI layers of RInChI layers two, three and four. The first letter of each group indicates the protonation state: “N” for zero in this example. Negative protonation states are indicated by stepping back through the alphabet (“M” for minus one, “L” for minus two, etc.) and positive protonation states by going on (“O” for plus one, “P” for plus two, etc.). The next four letters are generated by hashing the InChI minor layers for stereochemistry and other properties. For the reaction in Fig. 2, there is no stereochemistry and so these layers all are the blank “UHFF”.

The final three characters indicate the number of “no structure” components in layers two, three and four, respectively, with “Z” indicating zero such components, “A” indicating one, “B” two, etc. There is no check digit for the Short-RInChIKey.

#### Web-RInChIKey

The Web-RInChIKey is shorter still: only 47 characters. The brevity is useful, but, inevitably, means that more information is lost. On some occasions, the assignment of molecules to RInChI layers may be subjective. For example, water, in the reaction in Fig. 2, might be regarded as a reagent, and so in layer two or three, or else as an agent, and so in layer four. Similar reactions will then have different RInChI. The Web-RInChIKey ignores the distinction between layers two, three and four, and so the same Web-RInChIKey would be generated whether water is in layer four, or earlier in the RInChI.

The Web-RInChIKey for Fig. 2 is:

- Web-RInChIKey=SMUHAWIQPXIVCEVKG-NUHFFFADPSCTJSA

The first component is generated by combining InChI from all the layers of the RInChI and sorting them alphabetically. There should be no duplicates, but the program checks at this stage and removes any that are found. The major layers of all the InChI are then hashed to produce the first component (seventeen characters) of the Web-RInChIKey. The first character of the second component indicates the total protonation state, using the same code as for the Short-RInChIKey. In this example, the “N” indicates a total protonation state of zero. The next twelve characters come from a hash of the minor InChI layers. The final two characters, “SA”, again indicate the use of the Standard InChI and RInChI version “A”.

It is anticipated that the Web-RInChIKey will be widely used. Applications include searches over reaction databases with unknown drawing models, and comparison of reaction databases with different drawing models. The length of the strings for the major and minor layers should make searches over the Web rather precise.

## RAuxInfo

RInChI is based on the InChI of the components participating in the reaction. An InChI provides only the connectivity information of a molecule and so many different MOL files can be constructed from a single InChI, with different orientations or conformations of the molecule. This is an important feature of the InChI: molecules have the same InChI, whichever way up they are drawn. However, it can be useful to reconstruct the same orientation and conformation as was used to generate the InChI, and the data required for this is contained in the InChI-AuxInfo. Different representations of one molecule should always have the same InChI, but are likely to have different InChI-AuxInfo data.

In a similar way, it is not possible to go back from a RInChI to the original RXN/RD file, because the RInChI has deliberately discarded the formatting data. RAuxInfo (RInChI-AuxInfo) is a method of storing these data, which consists of four layers corresponding to the first four layers of RInChI. Layers 2, 3 and 4 of RAuxInfo are compiled from the AuxInfo strings of InChIs following the rules developed for RInChI using the order of the components determined for the RInChI calculation.

Reflecting the structure of the RInChI, the RAuxInfo data is presented in layers, separated by the same characters as the RInChI: “/”, “!”, “<>”. The first layer indicates the RInChI version and the InChI AuxInfo version: “RAuxInfo=1.00.1”. Layers two to four then list the InChI AuxInfo of the individual molecules in these layers, following the order of the RInChI with the same separators. The RAuxInfo for Fig. 2 is:
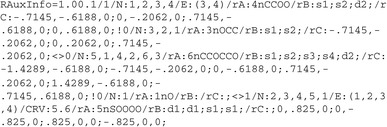


## Conversions

Whilst RInChI can be generated by hand, this is not a practical way of generating a significant database of reactions. Further, RInChIKeys cannot readily be calculated without a suitable computer program. The program can be downloaded from: http://www.inchi-trust.org/.

It is also available as an Oracle cartridge and as a Pipeline Pilot component from StructurePendium (http://structurependium.com).

### Generation of RInChI from RD and RXN files

RInChI V1.00 will take RXN files and RD files as an input and create RInChI, RAuxInfo and RInChIKeys from these. RD files separate starting materials and products. All molfiles outside the RXN sections, as well as catalysts and solvents, are interpreted as *agents*, present both before and after the reaction. RD files containing variations are not fully enumerated, and only the first entry is used to produce a RInChI. Multiple steps within a variation are summarized as a single step for one variation.

Neither RXN nor RD files can define reactions as equilibria or with unknown directionality, instead of starting materials going to a product. This information, if needed, has to be added separately to the RInChI. Multiple copies of a molecule should not appear within a RInChI, but the RXN or RD files can contain this information. In the current version, 1.00, there is no check for duplicate molecules, and so sometimes the same molecule will appear in more than one layer. It is intended to change this in future releases.

### Generation of RXN files from RInChI

Provided RAuxInfo is present, it is possible to generate an RXN file from an RInChI. In the absence of RAuxInfo, third-party software is needed to create suitable 2D coordinates for the molecules from the connection table stored in the InChI representation of individual molecules.

The use of the standard InChI means that stereocentres may not be handled consistently when rebuilt from RInChI without RAuxInfo. Reactions describing diastereoisomers may be rebuilt so that enantiomeric forms are displayed instead of a consistent stereochemical presentation.

## RInChI applications

### Generation of a RInChI for multistep reactions

For many processes, it is not clear whether they correspond to one reaction or to two or more successive reactions. RInChIs can be constructed for each step individually, and added together to express a multistep reaction as a single RInChI. For example, an aldol reaction is a very common transformation, involving an enolisation step and then a carbon–carbon bond-forming step. These may be two distinct steps, or it may be a continuous one-pot process. The RInChI for the two-step representation can be added to create a one-step representation. The first RInChI might be a ketone reacting with a base to make an enolate, and the second RInChI could be the reaction of the enolate with an aldehyde to form a β-hydroxy-ketone. The sum of the RInChI would be a new RInChI representing a ketone, base and aldehyde, reacting to form a β-hydroxy-ketone.

### Database analysis

A database of over a million RInChIs is available on: www-rinchi.ch.cam.ac.uk These data were extracted from patents by Daniel Lowe (NextMove Software). A million reactions cannot be analysed effectively just by reading through the list. The RInChI is a tool for identifying and extracting interesting features of the data. It is easy to gather statistics about common reagents and products. It can also find reactions with the same reactants and agents, but different products, thus quickly highlighting transformations that may deserve further study.

The Web-RInChIKey gives a rapid way of searching the database whilst ignoring the roles of the molecules involved. Reactions with the same Web-RInChIKey and yet different RInChIs and Short-RInChIKeys are easily identified as processes for which different studies have produced related but distinct data. Is a reagent present in the product, and so an agent, or did one of the reports mis-assign the role of a molecule? Gathering the relevant reaction reports makes it easy to answer such questions.

As multiple databases containing RInChI become available, this common label will enable consistent and straightforward analyses of diverse data.

### Consequences of the standard InChI

The RInChI uses the Standard InChI. This maximizes the consistency and interoperability of the RInChI, but has the consequence that some useful data are lost.

The Standard InChI describes absolute stereochemistry but does not describe relative stereochemistry. Many stereochemically-rich reactions are performed on racemic compounds. As a result, the RInChI is obliged to add absolute stereochemistry in an arbitrary way. Provided users are aware of the approach followed by the Standard InChI, we anticipate this will not lead to excessive confusion.

The Standard InChI also does some analysis and resolution of tautomers. For example, acetamide and acetimidic acid have the same Standard InChI, as do pyridin-2-ol and pyridin-2(1H)-one. For reactions in water, this is probably of no consequence. There may be situations, however, when it is useful for researchers to distinguish these substances and other related structures in reaction schemes. This is currently not possible using the Standard InChI.

In due course, if the InChI Trust recommends a new version of the Standard InChI, we expect that the standard RInChI would change to incorporate this new recommendation.

## Future

The InChI Trust has working groups developing many other aspects of the InChI standard, in order to extend its utility and to develop new applications. The RInChI is likely to interact with some of these developments. For example, a process for describing mixtures based on a similar approach to RInChI is under development (MInChI). It may be that details about reaction starting materials and products may be most conveniently recorded as MInChI. It could be of interest to apply both notations to describe multi-component systems [7].

There are also explorations of data analysis which use the RInChI and are complementary to the standard. For example, the Lapkin group at Cambridge [8] has proposed ProcAuxInfo. This is a method to record and structure detailed information from reaction processes: key data that is far beyond the reaction essence that the RInChI itself seeks to define, but which is needed by chemists and chemical engineers.

Currently, a RInChI can be converted back into a graphical representation only if AuxInfo has been supplied. Software is available which can generate clear molecular representations from InChI without AuxInfo and, in principle, the same thing can be done for RInChI. IUPAC recommendations on reaction layout may become available before too long. The conversion of RInChI to a clear and elegant graphical representation will remain outside the development of the RInChI itself.

The software currently creates RInChI from RXN and RD files. More opportunities for constructing RInChI from other data sources are under investigation.

## Conclusion

**Fig. 3:**
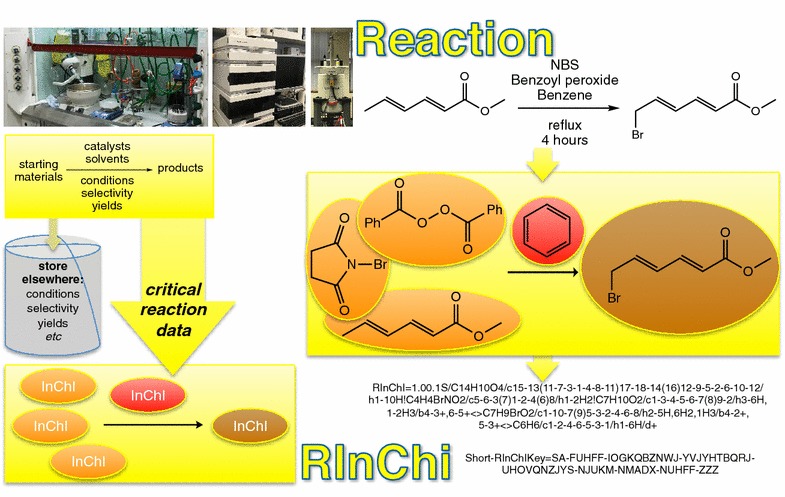
The RInChI Process

RInChIs provide a way of labeling reactions so that different people doing the same reaction should generate the same label (Fig. 3). This is a label that enables large databases to be searched quickly and consistently, and to discover new insights into chemistry. As more and more RInChIs become available, the more chemistry we will be able to discover and to understand.
